# Supplementary material for: Evaluating post 2024 election scenarios for the UK based on political party manifestos
Source: PLoS One. 2025 May 22;20(5):e0324942. doi: 10.1371/journal.pone.0324942 (PMC12097625; doi:10.1371/journal.pone.0324942)
Supplement: S3 File — (DOCX) [file pone.0324942.s003.docx]

Supplementary Material 3

| Scenario | Model implementation | Justification |
| --- | --- | --- |
| **Green Party** | Income Taxation= 3 | Additional tax for mid to high earners, and reform on capital gains tax |
|  | Green Taxation= 2 | Progressive increase in green tax |
|  | Windfall Taxation = 2 | Advocate for windfall taxes where there is evidence that market distortions are creating risk free additional profits. Initially retaining the levy on energy companies’ windfall profits and applying another on Banks following interest rates rises |
|  | VAT= -1 | Removed on some green items (e.g. solar on houses) |
|  | Green Jobs= 3 | Green skills and training of £4bn a year |
|  | Zero Hour= -1 | Rights for workers on zero hour contracts |
|  | Average Income= 2 | £15 minimum wage |
|  | Apprentices= 2 | Green skills and training of £4bn a year. £12bn over 5 years into lifelong training |
|  | Schools= 1 | £8bn for schools, £2bn of which is for teachers - possibly over 5 years. £5bn in special educational needs. £3bn for sixth form. |
|  | Universities= 2 | £30bn on research and development, not specifically university, for green funds across 5 years. Scrap tuition fees. |
|  | NHS= 4 | Extra £28bn a year by the end of parliament. Building repairs |
|  | Health & Wellbeing= 2 | Increase in public health budgets (restored to 2015 levels) and regulation of unhealthy products. Access to green space. Availability of healthy food |
|  | Social Care = 4 | Free social care. Investment of £20bn a year (50% increase) |
|  | Child care= 4 | 35 free hours a week. £1.4bn to sure start per year. |
|  | Crime= -1 | Different approach to crime than other parties. Academic evidence for these approaches, but lack of detail and numbers in this section of manifesto |
|  | Financial Markets= -3 | Significant greening and regulation of the sector. Do not support current 'fiscal rules' |
|  | Inequality= -2 | A maximum 10:1 pay ratio for all private- and public-sector organisations |
|  | Quantitative Easing= 2 | Not worried by fiscal rules |
|  | Private Investment= 2 | Cooperative development fund banks leading to investment in SMEs |
|  | Benefits= 3 | 5% increase in disability benefit. Lots of other increases. Plan for UBI |
|  | Carbon= -3 | Very large retrofit of homes. Measures to limit carbon impact of new homes. End to fossil fuel subsidies and new FF licences. 70% wind power by 2030. Renewable energy on homes. Frequent flyer levy and tax on aviation fuels |
|  | Biodiversity= 3 | Regenerative agriculture. Higher protection of Pas. NbS approach to rivers. Soil health programme. Access to nature. £3bn per year for rewilding |
|  | Pollution= -2 | Limited bans on single use plastic. Circular economy. Legislate lifespan of white goods. Clean air act. NbS approach to rivers. Extra funding for EA. |
|  | Agriculture= -1 | Higher welfare. Mainly on greening, so incorporated in other nodes. Likely slight reduction in output |
|  | Fishing= -2 | Destructive fishing banned from all waters |
|  | House Building= 3 | 150,000 social homes per year - equivalent to the current building of all homes |
|  | Road= -3 | £7bn in public transport. £6bn in active travel per year |
|  | Rail= 3 | £30bn a year |
|  | Water & Sewage= 3 | £12bn a year |
|  | Nationalism= 3 | Water and energy companies to be brought into public ownership |
|  | Judicial Changes= -1 | More leniency |
|  | Refugees= 2 | Safe routes and end of hostile environment |
|  | Legal Immigration= 3 | End of minimum income requirements. Simplified visas and ability to bring family members. An end to immigration detention for all migrants unless they are a danger to public safety. Those seeking asylum and protection to be permitted to work while their application is being decide |
|  | Foreign Aid= 3 | Restore to 0.7% then increase. Climate fund of 1.5%. Increase international aid to 1% of gross national income (GNI) by 2033.  Increase climate finance for the Global South to 1.5% of GNI by 2033, with an additional contribution to a newly established Loss and Damage Fund Continue to support Ukraine as it resists Russian invasion |
|  |  |  |
| **Reform UK** | Income Taxation= -2 | Increase non-taxable threshold to £20k. 40% starts at £70k. Introduce a UK 25% transferable marriage tax allowance. Scrap Interest on Student Loans. Residential Stamp Duty to 0% below £750k; 2% from £750k - £1.5m; 4% over £1.5m. Abolish Inheritance Tax, IHT, for all Estates under £2m. The rate above £2m will be 20% tax, with the option to donate to charity instead. |
|  | Green Taxation= -3 | Scrap Annual £10bn of Renewable Energy Subsidies. Cut Energy Taxes. Lower fuel duty by 20p per litre. Scrap environmental levies. |
|  | Corporate Taxation= -2 | Lift the minimum profit threshold to £100k. Reduce the main Corporation Tax Rate from 25% to 20%, then to 15% from year 3. Lift the VAT Threshold to £150k. Abolish Business Rates for High Street Based Small & Medium Firms. Cut entrepreneur’s tax relief to 5%. SME Enterprise Zones for left-behind Areas with a period of zero tax for new or existing businesses that are creating jobs. |
|  | VAT= -2 | Abolish the VAT Tourist Tax. Lift the VAT Threshold to £150k. Tax relief of 20% on all Independent Education. Scrap VAT on energy bills |
|  | Employment= 1 | Abolishing IR35 rules. Enforce a 2-Strike rule for job offers. |
|  | Public Sector Pay= -1 | Save £5 in every £100. Every manager across government must find savings without touching frontline services |
|  | Green Jobs= -3 | Scrap all Net Zero related objectives and subsidies. Scrap climate-related farming subsidies. Scrap Annual £10bn of Renewable Energy Subsidies. |
|  | Zero Hour= 2 | Cut government regulations and "nanny" state. |
|  | Apprentices= 2 | Tax relief for businesses that undertake apprenticeships. |
|  | Universities= -3 | Cut funding to universities that undermine free speech. Restrict undergraduate numbers well below current level. Introduce new visa rules for international students and their dependents. Scrap Interest on Student Loans. Enforce minimum entry standards. 2 year degrees. |
|  | Skills Gaps= -1 | Smart immigration to target essential skills in healthcare only. New apprenticeships and vocational courses to replace cheap overseas labour. |
|  | NHS= 1 | All frontline NHS and social care staff to pay zero basic rate tax for 3 years. Cut waiting lists by using UK & overseas providers. Cut waiting times with a campaign of ‘Pharmacy First, GP Second, A&E Last’. End training caps for all UK medical students. |
|  | Health & Wellbeing= -1 | Withdrawing benefits if no job acceptance after 2 offers. Abolish renters reform bill. Launch inquiry into Social Media Harms. Review the Online Safety Bill. |
|  | Social Care= -1 | Commence Royal Commission of Inquiry into Social Care System Reform Social Housing Law. All frontline NHS and social care staff to pay zero basic rate tax for 3 years |
|  | Private Health Care= 3 | 20% tax relief on all private health care and insurance. Patients receiving vouchers for private treatment if they can't see a GP in three days. |
|  | Crime= -2 | More investment in detention centres. Commence building of 10,000 New Detention Places. Prison for all violent crimes and possessing a knife. |
|  | Inequality= 1 | 40% tax starts from £70k. Tax cuts for independent schools and private health care. IHT from £325k to £2 million. Scrap all Diversity Equality and Inclusion roles. Mandate Single Sex Spaces. Propose a Comprehensive Free Speech Bill. |
|  | Quantitative Easing= -2 | Reform Party is against this. This saves £35 bn per year on interest payments. |
|  | Private Investment= 2 | Tax reliefs and removing laws. |
|  | Benefits= -2 | All Job seekers and those fit to work must find employment within 4 months or accept a job after 2 offers, otherwise benefits are withdrawn. |
|  | Cost of Living= -1 | Scrap VAT on energy bills. Lower fuel duty by 20p per litre. Scrap environmental levies. |
|  | Treasury Funds= 1 | Over £140bn planned savings through cutting taxes, benefits, foreign aid (by 50%) and government departments. Increase budget for both the National Crime Agency and The National Drugs Intelligence Unit. Increase Defence Spending to 2.5% of National GDP by year 3. Bank of England Must Stop Paying Interest to Commercial Banks on QE Reserves |
|  | Carbon= 4 | Start fast-track licences of North Sea gas and oil. Grant shale gas licences on test sites for 2 years. Scrap environmental levies. Scrap bans on selling petrol and diesel cars and legal requirements for manufacturers to sell electric cars. Abandon Net Zero. Scrap climate-related farming subsidies. |
|  | Biodiversity= -3 | Productive land must be farmed, not be used for solar farms or rewilding. |
|  | Pollution= 2 | Scrap environmental levies. |
|  | Agriculture = 2 | Increase the farming budget to £3bn. Taxpayer funded organisations should source 75% of their food from the UK. Help farmers sell their produce directly to the public. Change planning laws to support farm shops with zero business rates. |
|  | Fishing = 2 | Tax and other incentives to ensure that all fish caught in British waters are landed and processed in the UK. Tax incentives and vocational training to increase UK fishing fleets |
|  | House Building = 2 | Fast track planning and tax incentives for development of brownfield sites. Review planning system. |
|  | Road = 3 | Lowering fuel duty. Ban ULEZ and low traffic neighbourhoods. Accelerate transport infrastructure including roads. |
|  | Rail = -1 | Scrap HS2. |
|  | Judicial Changes = 3 | Withdraw citizenship from immigrants who commit significant crimes. Change the definition of Hate Crime. Leave the European Convention on Human Rights. Automatic life imprisonment for violent repeat offenders. Commence Reform of the House of Lords. Commence Reform of the Civil Service. |
|  | Police= 3 | Increase UK per capita police numbers to 300 per 100k population. Allow PCSOs to become police officers before the role is phased out. Replace degree-standard entry with an entrance exam. |
|  | National Security= 2 | Introduce incentives and tax breaks to boost the UK defence industry. Improve equipment self-sufficiency and manufacture world class products for export. Increase Defence Spending to 2.5% of National GDP by year 3, then 3% within 6 years. Increase basic pay across our armed forces to boost recruitment and retention. Introduce a new Armed Forces Justice Bill. Education for Military Personnel. Expand the Royal Navy Overseas Patrol Squadron |
|  | Refugees= -2 | Stop the Boats with 4 Point Plan. Leave the European Convention on Human Rights; zero illegal immigrants to be resettled in the UK; possible offshore processing for illegal arrivals; secure detention for all asylum seekers. Pick up migrants and return to France. |
|  | Legal Immigration= -2 | Freeze Non-Essential Immigration. New Department of Immigration. Only international students with essential skills can remain in the UK when their study ends. |
|  | Foreign Aid= -3 | Cut Foreign Aid by 50%. |
|  |  |  |
| **Liberal Democrat** | Income Taxation= -2 | Raise the tax-free personal allowance. |
|  | Corporate Taxation= 2 | Tackle international corporate tax avoidance. Make the case for increasing the global minimum rate of corporation tax to 21%. Reverse tax cuts for banks. Digital services tax. Share buy-back. |
|  | Windfall Taxation= 2 | Implementing a proper, one-off windfall tax on the super-profits of oil and gas producers and traders. |
|  | Zero Hour = -2 | Right to request a fixed-hours contract after 12 months for ‘zero hours’ and agency workers, not to be unreasonably refused. |
|  | Apprentices= 3 | Investment in more apprenticeships. New Lifelong Skills Grants for adults to spend on education and training throughout their lives. |
|  | Schools = 2 | Mental health professionals in schools. Investing in buildings and pupil premium. |
|  | Universities= 2 | Ensuring universities widen participation by disadvantaged and underrepresented groups. Requiring universities to be transparent about selection criteria. 3% of GDP into Research. Links to more European funding. |
|  | NHS= 2 | 8000 new GPs (~20%), dentists (undefined amount), and mental health hubs (undefined). |
|  | Health & Wellbeing= 2 | Expanding early access to health services. Vape ban, taxes on unhealthy foods |
|  | Social Care= 2 | Trialling personal health and social care budgets so that individuals are in control of what care they receive. Extra £2 an hour to social care workers minimum wage. Carers allowance increase. |
|  | Private Health Care= -1 | Bringing dentists back to the NHS from the private sector by fixing the NHS dental contract. |
|  | Child Care= 1 | Replacing the three different current registration processes with a single childcare register. Early months maternity/ paternity increase |
|  | Private Investment = 2 | Expand the British Business Bank to perform a more central role in the economy, to ensure that viable small and medium-sized businesses have access to capital, and enable it to help ‘crowd-in’ private investment. Relationship with Europe to allow more investment. |
|  | Pensions =3 | Measures to end the gender pension gap in private pensions and ensure working-age carers can save properly for retirement. Protect the triple lock so that pensions always rise in line with inflation, wages or 2.5% – whichever is highest. |
|  | Benefits = 2 | Social tariff for energy. Removal of 2 child cap. Annual increases to universal credit. |
|  | Carbon = -3 | Restoring peatlands, banning use of horticultural peat and the routine burning of heather on peatlands. Make it easy and cheap to charge electric vehicles. Invest in green infrastructure and home energy upgrades. Decentralisation of power generation. Pressing for the ending of fossil fuel subsidies internationally. Carbon reduction road map for industry and stock market companies. Tree planting. regulation of blue and agri carbon markets. |
|  | Biodiversity= 4 | Ensuring new developments result in significant net gain for biodiversity, with up to a 100% net gain for large developments. Restoring peatlands and other NbS. Doubling the size of the protected area network. Tree planting - 1% of the UK per year. |
|  | Pollution = -3 | Cut resource use, waste and pollution by maximising the recovery, reuse, recycling and remanufacturing of products. Regulation of water and regulatory bodies (OEP). |
|  | Agriculture= 1 | Measures to increase welfare more than amount of food produced. Little change to the amount of farming, but more profitable. |
|  | Fishing = -1 | Rebuilding depleted fish stocks to achieve their former abundance, ban on bottom trawling in marine protected areas. Fishers, scientists and conservationists at the centre of the regionalised fisheries management system. |
|  | House Building = 4 | Increasing building of new homes to 380,000 a year, including 150,000 social homes. |
|  | Road = -2 | Support rural bus services and encourage alternatives to conventional bus services where they are not viable.  Maintain the £2 cap on bus fares while fares are reviewed. Make it easy and cheap to charge electric vehicles. |
|  | Rail = 3 | Make rail convenient, affordable and environmentally-friendly for passengers and freight. Investment, but not quantified |
|  | Water & Sewage = 4 | Banning water companies from dumping raw sewage into rivers, lakes and coastal areas. End the sewage scandal by transforming water companies into public benefit companies |
|  | Nationalisation = 1 | Water as public benefit companies |
|  | Police = 1 | Freeing up police time, reducing court backlogs. Some commitment to increase numbers - not quantified. |
|  | National Security= 2 | Increase military troop numbers back to 100,000, Funding for NATO. |
|  | Refugees= 1 | Work across borders to provide safe and legal routes for refugees and tackle common threats such as human trafficking, cybercrime and terrorism. |
|  | Legal Immigration = 2 | Measures to increase legal immigration for the NHS and other skilled workers. |
|  | Foreign Aid = 1 | 0.7% of GDP to national development - with focus on climate |
|  |  |  |
| **Conservative** | Income Taxation= -1 | 2p off National Insurance, and further cuts for self employed. No increase in income tax or VAT. |
|  | Green Taxation = -1 | No new green levies. Removal of some. |
|  | Windfall Taxation= 1 | Continuation of windfall tax on oil and gas companies until 2028-29, unless prices fall back to normal sooner. |
|  | VAT= -1 | Keep the VAT threshold under review |
|  | Average Income= 1 | Rise in living wage |
|  | Apprentices= 2 | Creation of 100,000 more apprenticeships |
|  | Schools= 1 | Protection of the current level of spending. Bonuses to STEM teachers. 60,000 new school places |
|  | Universities= -2 | Closure of poor performing courses. £22bn on research and development, university funding not specified within this |
|  | Skills Gaps= -2 | We will deliver the Lifelong Learning Entitlement, giving adults the support they need to train, retrain and upskill flexibly throughout their working lives. From the 2025 academic year, adults will be able to apply for loans to cover new qualifications. We will also continue to expand our adult skills programmes, such as Skills Bootcamps which meet skills shortages. |
|  | NHS= 2 | 92,000 more nurses and 28,000 more doctors in the NHS than in 2023. Dental Recovery Plan will unlock 2.5 million more NHS dental appointments. 40 new hospitals by 2030. Maternity and mental health improvements. |
|  | Social Care= 2 | We are committed to supporting a high-quality and sustainable social care system |
|  | Child Care= 3 | Give working parents 30 hours of free childcare a week. |
|  | Crime= -1 | toughen sentences for knife crime, grooming gangs and assaults against retail workers. |
|  | Private Investment= 1 | Deliver tree planting and peatland commitments through Nature for Climate funding, and continuing our work to unlock private investment. Working with British Business bank for women entrepreneurs. £250m, but not assured. |
|  | Pensions= 2 | Remove tax on pensions. £430 a year rise. |
|  | Benefits = -2 | Child benefit increases (~£1500 per year per family). Suite of reforms to make benefit claiming harder. Tighten up how the benefits system assesses capability for work. |
|  | Treasury Funds= 1 | Savings from cutting civil service (£20bn estimate) |
|  | Carbon= 1 | Build the first two carbon capture and storage clusters. Deliver current tree and peat strategies. EV charging stations. Support North Sea oil. New gas power. Treble offshore wind. Scale up nuclear power. Carbon pricing scheme for steel etc. |
|  | Pollution = 3 | Reversing the London Mayor’s ULEZ expansion |
|  | Agriculture =2 | Continue to ringfence agricultural funding so it is passed directly on to farming and rural communities in Scotland, Wales and Northern Ireland alongside a new UK-wide £20 million Farming Innovation Fund. £1bn investment over 5 years. |
|  | Fishing= 1 | Creating new fishing opportunities. £100 million seafood fund |
|  | House Building = 4 | Deliver 1.6 million homes in England, increase in stamp duty |
|  | Road= 3 | £36bn in road and rail, most on road, 20% ish on buses. £8.3bn to fill potholes and resurface roads, funded by the cancellation of railway development (HS2). Reverse ULEZ. |
|  | Rail = 1 | Upgrades to railways estimated at £14bn, funded by scrapping of HS2 |
|  | Judicial Changes= 2 | Amend the law so judicial reviews that don’t have merit do not waste court time |
|  | Police =1 | 8,000 more officers |
|  | National Security= 2 | Boost in defence spending to the new NATO standard of 2.5% of GDP by 2030 |
|  | Refugees= -1 | Stop the boats by removing illegal migrants to Rwanda. Reford asylum treaties |
|  | Legal Immigration= -2 | Legal cap on migration to guarantee that numbers will fall every year. |
|  | National Service = 2 | Introduce mandatory National Service for all school leavers at 18, military or civic service roles. |
|  |  |  |
| **Labour** | Income Taxation = 1 | Remove non-dom status tax avoidance measures |
|  | Corporate Taxation= -1 | Capped at 25% |
|  | Windfall Taxation= 3 | Close loopholes in windfall tax on oil & gas companies. Increase in the rate of levy by 3 percentage points and remove unjustifiable investment allowances. |
|  | Employment= 1 | Bring Jobcentre Plus and National Careers Service together to provide national jobs. Tackle backlog of ‘access to work’ claims. |
|  | Green Jobs= 3 | ‘Make Britain a clean energy superpower’ main mission= 650,000 new high-quality jobs; new nuclear power stations, such as Sizewell C, and Small Modular Reactors. Reward clean energy developers with a British Jobs Bonus- £500m per year from 2026, to incentivise firms who offer good jobs. Carbon capture and green hydrogen investments |
|  | Zero Hour= -4 | Banned |
|  | Average Income= 1 | Minimum wage increases. Remove discriminatory age bans so all adults are entitled to the same minimum wage. |
|  | Apprentices = 3 | A youth guarantee of access to training and apprenticeships or support to find work for all 18-21 year olds |
|  | Schools= 2 | 9,500 more teachers (1.9%)  Breakfast clubs and mental health professionals |
|  | Universities= 1 | Longer term funding for research. Support for spinouts. |
|  | NHS= 1 | 40,000 extra appointments per week. Modernise facilities. Link with social care. 700,000 dentist appointments. 8,500 mental health staff. |
|  | Health & Wellbeing = 2 | Mental health professionals in schools. Ban/ restrict smoking and junk food products. |
|  | Social Care = 1 | Strengthen Stalking Protection Orders. Introduce domestic abuse experts in 999 call centres. Introduce disability and ethnicity pay gap reporting for large employers. |
|  | Youth Clubs= 2 | Young Futures Programme to every community. |
|  | Private Health Care = 1 | Use of private sector to reduce NHS waiting lists |
|  | Child Care = 2 | 3,000 more nurseries |
|  | Crime = -2 | Greater focus on prevention. Ensure those financially benefiting from knife crime are being held accountable. Introduce new Respect Orders to ban persistent adult offenders from town centres. Create new specific offence for assault on shop workers. |
|  | Economic Growth= 1 | ‘Kick-start economic growth’ main mission = make current budget moves in balance and ensure debt to fall. Labour will strengthen the role of the Office for Budget Responsibility |
|  | Financial Markets= 2 | Green Investment Hub & OBR regulatory changes to improve stability |
|  | Inequality= -2 | School breakfast clubs. Social legislation on discrimination. Protect renters from arbitrary eviction. Ban zero hour contracts. |
|  | Private Investment = 4 | The National Wealth Fund will target 3:1 return on £7.3 billion. £500 million to incentivise green jobs in deprived areas |
|  | Pensions= 1 | Increase investment from pension funds in UK markets to deliver better returns. Transfer Investment Reserve Fund back to members (mine workers). |
|  | Cost of Living= -2 | Make housing more affordable. Expand access to child care. Keep mortgage rates as low as possible. Free breakfast clubs in every primary school. Invest £6.6 billion to upgrade 5 million homes to cut bills. |
|  | Treasury Funds= 1 | Various revenue schemes such as VAT on private schools |
|  | Carbon = -2 | Carbon capture and Green Hydrogen- Investment of £1.5 billion. Double onshore wind. Triple solar power. Quadruple offshore wind by 2030. |
|  | Biodiversity = 1 | Promote nature recovery, new forests and expansion of peat bogs. |
|  | Agriculture = 1 | Champion British Farming. Introduce a Land Use Framework. |
|  | House Building = 4 | 1.5 million new homes over 5 years (currently 150,000 a year) |
|  | Road = 3 | Changes in planning to build new roads.  Reduce car insurance. Commitment to reform bus franchises. |
|  | Rail = 2 | Better Northern Connectivity. 10 Year infrastructure strategy. |
|  | Water & Sewage = 1 | Reservoir commitments.  Give regulators new powers to block the payment of bonuses to polluting executives. |
|  | Nationalisation = 2 | Nationalisation of railways and £8.3 billion into energy. |
|  | Judicial Changes= 3 | ‘Take Back Our Streets’ main mission- fast track rape cases with specialised courts, introduce new protection of victims of crime. |
|  | Police = 2 | £130,000 in costings. Thousands of extra officers. Changes to police power over knives and anti-social behaviour. Rape and domestic abuse support. |
|  | National Security = 2 | 2.5% of GDP on defence. Commitment to UK’s nuclear deterrent. Bring in Martyn’s Law to strengthen the security of public events. |
|  | Refugees= -2 | Create a new Border Security Command. Seek a new security agreement with the EU for joint investigations. Hire additional caseworkers to fast track removals to safe countries |
|  | Legal Immigration= -2 | Reform the points-based immigration system. Appropriate restrictions of visas. Link immigration and skills. Employers who break rules will be barred from employing workers from abroad. |
|  | Foreign Aid = 1 | Commitment to Nato and our nuclear deterrent. Development spending to 0.7% GDP |
