# Supplementary material for: Evaluating post 2024 election scenarios for the UK based on political party manifestos
Source: PLoS One. 2025 May 22;20(5):e0324942. doi: 10.1371/journal.pone.0324942 (PMC12097625; doi:10.1371/journal.pone.0324942)
Supplement: Attachment — Submitted filename: Response to Reviewers.docx [file pone.0324942.s005.docx]

**Response to Reviewers**

*We would like to thank all the reviewers for their time and effort in reviewing the paper. Overall, it is clear that the purpose of the study has not come across clearly, and we have tried to address this in a newly written introduction section. Hopefully the aims and purpose of the study are much clearer (we especially thank reviewer 2 for their suggestions, and have incorporated these into the new aims). It was also clear the methods were not as clear as they could be. We have rewritten this section, but we also refer to a recently published ‘methods’ paper (by a subsection of the authors of this paper) on the BBN package used, which also provides more details. We have also reorganised the combined results and discussion into separate sections, to allow the study’s aims to be more fully discussed, and not just a party by party outcome of the results. We have also removed the second ‘environmental model’ from the analysis, as we felt this added little to the scope of the paper, and didn’t fit clearly with the new aims.*

*We have addressed comments one by one – but due to substantial rewriting of the manuscript, it is hard to provide quotations or line numbers of exactly what has been changed, and the rewriting has also meant the ‘track change’ manuscript is difficult to follow. Our responses are in italics.*

Reviewer #1: This paper seeks to evaluate the post-2024 election scenarios based on the UK party manifestos. The exercise is interesting and the authors develop a sophisticated model, but it has several major problems that make it unsuitable for publication in an academic journal. I list below my main concerns.

The first major problem is that it is not obvious what we learn from this exercise. It is interesting to derive models out of manifestos, but it is not clear why we need to know this. After reading the paper, I had a major question mark: “so what?”. Predicting how policies would look like may be interesting, but experience showed that such predictions are very often wrong and they do not match reality. We know from existing studies that a) campaign promises are not always feasible and b) circumstances change during a term in office – the dynamic of politics and society cannot be anticipated based on manifestos. In brief, it is not obvious what we gain after reading these predictions in addition to simply reading the manifestos. More important, the manifestos are programmatic promises that are rarely accomplished; in many democratic countries, political parties fulfil a small share of their manifesto promises, which means that such predictions lose their purpose entirely.

*We have clarified the contribution of the paper in the introduction and discussion. While it is true that commitments in manifestos may never materialise in full in government (we have added to the introduction to illustrate this), we argue that the manifesto is the best approach we have to knowing which policies will be enacted by that party, if it is in government. We explain that manifestos have policy levers and aspirational outcomes, but due to the wider political landscape being a complex system, it is not possible to imply cause and effect as simply as manifestos do. As such we identify three aims, where we assess: (1) the effectiveness of policies in political party manifestos in achieving societal, environmental and economic outcomes, (2) unintended or second order consequences of policies, and (3) create a prediction of the overview of the holistic outcomes different manifestos would achieve, if enacted in full. We demonstrate how the model helps achieve all of these aims.*

One implicit assumption of the models is that the situation during the term in office will be similar to that during the campaign in which these promises are formulated. There are many studies indicating that this is not the case, and much changes. Neither endogenous nor exogenous sources of change are included in the discussion. Moreover, even if we assume that everything stays the same in terms of the context, how do we know that the actors’ preferences do not change based on new information that they acquire or on new opportunities? The model does not account for these changes. Also, the model does not consider possibilities for coalitions or changing government partners.

*This is true, and literature on these points has been added to the introduction. However, while we acknowledge these weaknesses, these are not the aim of the study, which we indicate above. Voters need to make decisions based on manifestos (or reporting of manifestos in media), and our models provide more information on the holistic actions of a party’s intended policies at the time of voting.*

Another major concern is that the manuscript does not engage with the vast literature on pledge fulfilment or strategic role of manifestos. Taking at face value the content of manifestos is not common and extensive literature shows that manifestos play a political communication role in many instances rather than aspiring to pursue specific policies.

*We acknowledge this, and have added a section to the introduction about pledge fulfilment. The latter point is also addressed, we pull out specific and quantifiable policies from the manifestos to predict the aspirational outcomes also listed in the manifesto. These outcomes are not taken at face value.*

The 2024 elections are a bad choice in terms of programmatic policies because there was a clear winner and a major loser no matter what they included in the manifestos. These are atypical elections, which were called very shorty before the date in which they were organized, parties did not have time to prepare their programmatic promises and thus there is limited credibility regarding the pursuit of some policies.

*The fact that there was a clear winner and loser does not seem relevant to this work. We do appreciate the election was called early, however, all parties did know there was an election occurring in 2024 and would have been preparing policies. Again, the manifestos provided the information that voters needed to make decisions on.*

Finally, the paper does not have an analytical goal and it is mainly a description. This is useful for a course session or for journalistic accounts, but insufficient for a journal article.

*We have added a clearer set of aims to the paper, and discussed the results in light of these aims.*

Reviewer #2: This was an interesting paper and potentially a very significant contribution to the academic literature on party politics and the study of party programmes i.e. manifestos, in particular. In my view, it meets four of the journal's criteria: it presents the results of primary research, I'm not aware that the results have been published elsewhere, it meets the standards of research integrity and it adheres to community for standards for availability. It is also partially meets the remaining criteria: it is performed to a high technical standard (although there is work to be on clearly describing the approach); the conclusions are supported by the data (but the conclusions are also pointing in the wrong direction); and the article is generally clear, but it could also be much clearer.

Overall, I think this paper needs to be revised before it should be published. However, I think it should be revised for very positive reasons. I'm someone who researches manifestos, but the approach taken in this paper was extremely original. I think the authors do not do enough to capitalize on the paper's significance, and I strongly, strongly encourage them to do so. The paper should should be read and considered by all students of party politics, political campaigning, and party manifestos/platforms. Crucially, I think it has the potential to reach that audience.

[1] The paper could be very much stronger if it were reframed. It is currently framed as an almost bizarre counter-factual: what would have happened if party X, Y or Z had been elected and implemented its manifesto? This current framing is intellectually unsatisfying and further lacks realism (e.g. the way the UK system works, the importance of sequencing in the legislative timetable, the effect of events). To my mind, the obvious solution is to frame this paper about pledges and the fact that party pledges have second-order consequences on others. All studies of manifesto pledges and party ideology based on manifestos consider them narrowly in terms of their first-order effects. But this paper makes a very good case to re-think that. THIS IS VERY IMPORTANT!!! (Please excuse the emphasis...) A way to do this would be along the lines of: "Studies of manifestos pledges consider simply whether they were fulfilled or not. There is also a tendency to think about policy proposals in isolation rather than as part of joined-up programmes. This paper demonstrates the importance of joining up pledges to consider the internal consistency of manifesto commitments." I think such a framing would guarantee very high citation rates in Pol Sci journals.

*We thank the reviewer for the suggestion. We have re-written the introduction to frame the work differently. We discuss the difference between policy levers governments have complete control over, and aspirational outcomes, and centre the study around three aims: (1) the effectiveness of policies in political party manifestos in achieving societal, environmental and economic outcomes, (2) unintended or second order consequences of policies, and (3) create a prediction of the overview of the holistic outcomes different manifestos would achieve, if enacted in full.*

[2] Related, the paper needs to do more to engage with the way political scientists have studied manifestos. Broadly speaking, there are two approaches: the pledge-fulfillment literature, and the spatial positioning of parties (i.e. the Manifesto Project). There needs to be some attempt to ground the paper in these literatures, even if the current framing is retained. Ideally, this literature would be a clearer jumping off point for the research (as per point 1).

*We have added a short section on fulfilment of pledges to the introduction of the paper. We do acknowledge pledges are often not fulfilled, at least in full, but as indicated elsewhere in this response, we want to focus the study on how the model provides information on outcomes, based on policy which* can *be enacted. We use some of our data to examine spatial positioning more in the revised discussion.*

[3] I was slightly discomfited by the decision to stick with draft proposals (Reform UK) for the use of quotes. That doesn't come across as well as it could. If the research is worth doing, it's worth doing fully. I'm also not convinced by the decision to exclude the SNP and PC, especially if the goal is to consider the second-order consequences of proposals. Remember also that the Scottish Greens are a separate party from the England and Wales Green Party. The latter do not in Scotland.

*We fully agree with this point about the draft manifesto. The reason was the preprint of this paper appeared before the election, and the final version of the Reform manifesto was released late. We did check the final version would not greatly influence our results. However, for this revision, we have changed the paper to reflect to Reform manifesto – one quote from within the paper was only present in the draft manifesto, and we have kept this, but in terms of the analysis we have recoded supplementary material 3 and re-analysed the results. There are only a few subtle differences in the results, compared to before.*

*We did also consider the inclusion of other parties (SNP and Plyd Cymru in particular). Ultimately, more parties makes the analysis of the paper more complex, and we decided to stick to the five main English parties (even though some of these will have the same manifesto for the devolved nations). We have added text to the methods to justify this point.*

[4] Going back to point 1 and the counter-factual framing of the paper, one obvious validity test of the current approach is to consider the actual implementation of the 2019 Conservative manifesto on the environment. Did the estimate from 2019 bear closer resemblance to the actual performance of the Johnson-Truss-Sunak government? Alternatively, if these things cannot be ascertained, we're back to what the paper can get at with greater validity: the second-order impact of manifesto pledges.

*Unfortunately the environmental pledges of the last government shifted enormously through their term in office. This is likely far more than normal, partly due to Covid as a major external disruption, partly due to three different prime ministers. We hope the new framing of the paper, and the removal of the environmental model, for the sake of simplicity, helps address this point.*

[5] The paper needs to be much clearer around the methods employed. I found it difficult to follow, for example, whether the coding was computer-aided or manual, or how the scoring was arrived at. For this paper to have the reach it could potentially have, it needs to be written for those who study parties and won't have a clue about the modelling being done here. Basically, make sure the methods are written in a way that would enable, say, a historian to follow the logic.

*We hope we have addressed this. We have published a methods paper for the technique and do refer to this for some of the technical details. We have largely divided the methods into different sections, some of which are more technical than others – but it should be possible to understand the principles of the model if not a specialist in this area. We have also clarified the approach to parametrising the model, and added a section on subjectivity of the models to the methods.*

Those are really my main thoughts. In essence: this is potentially very impactful - but it could usefully be reframed and made more accessible. I would encourage the authors to do so in for this research to fulfil the potential I think it has.

Reviewer #3: This paper aims to evaluate party manifestos in the UK 2024 general election and predict the potential policy outcomes if the manifesto promises were realized. However, several fundamental concerns make it difficult for me to fully appreciate the value of this study.

The model construction lacks transparency. The coding process appears highly subjective. The authors did not provide detailed rationales for how the edges and edge strengths were coded, nor did they explain how the quality of the coding could be evaluated. For example, do the authors assess intercoder reliability?

*We have rewritten much of the methods to address this, including a clearer response to* *parametrising the model, and added a section on subjectivity of the models to the methods.*

The authors fail to consider the underlying motivations of the parties in making their policy promises. Depending on the type of party, there are different reasons for raising certain issues in their manifestos, and these should not be treated equally.

*As indicated above, our analysis focusses on policy levers available to governments. It then assesses the real outcomes of these actions against what popular opinion may be. In our revised introduction we do acknowledge and discuss how manifestos also specify outcomes, which may not result from their policies. Furthermore, given the framework we have partially adopted from reviewer 2, we have a focus on whether policies have unintended consequences on outcomes. However, we do acknowledge we are taking direct policy levers at face value, and these may never be fulfilled. But we are predicting the outcomes of these levers, which will potentially highlight motivations of some of these actions.*

The political context and process are completely ignored in the analysis.

*We don’t really understand what this comment is getting at.*

Overall, it is challenging to discern what meaningful insights can be gained from this analysis.

*We hope the revised manuscript addresses the concerns.*

Reviewer #4: The comments for Author are as under:
1. The introduction is informative and well-organized, but it would benefit from a clearer articulation of the study's primary research question. While the scope of the work is detailed, explicitly stating the research objectives upfront would help frame the analysis more effectively for readers.

*We hope this has been achieved*

2. While the paper references key literature on complex systems, political science, and Bayesian belief networks (BBNs), there is room for improvement in establishing a more robust link between previous research and the current study. Consider expanding the literature review to include more recent studies and critical perspectives that contextualize the use of BBNs in political scenarios.

*We have added a few references and points to the introduction, however, while the complex system approach has been suggested previously, and many seem to agree, given the citations of some of the key papers, little direct work has been done with BBNs in this context. We have made mention to similar studies, where this has been possible.*

4. The methodology is thorough, but more clarity is needed regarding the model’s assumptions. Specifically, providing a more detailed justification for the selection of node strengths and the interpretation of feedback loops within the Bayesian network would enhance the reader’s understanding of the modeling process. Additionally, the rationale for excluding smaller, devolved political parties should be more clearly addressed.

*We have updated the methods (see above) and hopefully this is clearer. We do refer to a methods paper on BBN recently published, which provides more detail of the underlying equations etc. With the refocus of the paper (following partly from reviewer 2’s suggestions) we have decided not to include additional parties, simply due to the complexity of comparing results, and the five parties studied provide plenty of evidence to address the study aims.*

5. The results section presents valuable insights but could benefit from a more in-depth discussion on the potential limitations of the predictions. For example, consider addressing the risks associated with using simplified models for complex political scenarios. Discuss how uncertainties and assumptions in the model might affect the real-world applicability of your findings

*We have added a section to the results and discussion section.*

6. Overall, the manuscript is well-written, but there are occasional grammatical errors and awkward phrasing. A thorough proofreading is recommended. The paper maintains an appropriate academic tone but occasionally lapses into informal language. Ensure that all sections adhere to formal academic writing standards

*We have reread the paper and much of it has been rewritten and restructured*

7. Application of Bayesian belief networks to evaluate political manifestos and future policy outcomes. However, to enhance its contribution, the authors could discuss how this approach could be applied in other countries or in forecasting long-term political outcomes beyond the immediate post-election period. This would add greater depth to the potential broader impact of the study.

*We have added this to the end of the discussion section, including how this approach can highlight the outcomes of popularist parties, and illustrate why some policies such as tax cuts may not be good for the majority of voters.*
