# Supplementary material for: Evaluating post 2024 election scenarios for the UK based on political party manifestos
Source: PLoS One. 2025 May 22;20(5):e0324942. doi: 10.1371/journal.pone.0324942 (PMC12097625; doi:10.1371/journal.pone.0324942)
Supplement: Attachment — Submitted filename: Response_To_Reviewers_auresp_2.docx [file pone.0324942.s006.docx]

Reviewer #3: The authors' response to the reviewers' comments was not comprehensive, which is understandable given the fundamental nature of some of the issues raised. While the model presents an interesting intellectual exercise, its development still appears pretty subjective in the revised version. This makes it difficult to convincingly demonstrate the scientific contribution of the paper.

*Any predictive model of this scale would have some degree of subjectivity. We argue that the approach we have taken, using a simple framework largely free of complex equations to model a complex system, and a multi-author group to discuss and agree final parameters of the model not only helps eliminate individual subjectivity, but also provides a transparent framework of the modelling assumptions made, which is fully scrutinisable (and reproduceable following comments from reviewer 5, below) by a wide variety of academics and other interested parties, including those with limited mathematical modelling experience.*

Moreover, the authors treat the manifestos of all parties equally, failing to recognize the significant differences between potential ruling parties and fringe parties. Potential ruling parties may view manifestos as tools for signaling policy intentions to voters and potential coalition partners, while fringe parties may primarily use them to advance special interests or raise awareness of their positions. These differing motivations, along with the political context surrounding an election and the anticipated interactions among political actors, likely influence how parties strategically craft their manifestos. Furthermore, manifestos can be seen as initial proposals for a bargaining process, with final policies reflecting negotiations and compromises among various actors. Given these complexities, the model's assumption that a party can be the sole ruling party raises concerns about the generalizability and interpretability of the results. It remains unclear what insights can be gleaned from the analysis without considering the strategic dynamics of coalition formation and policy bargaining.

*We don’t agree with this comment. Both voters and journalists use policies and pledges within manifestos in their writing and decision-making processes. We appreciate that different parties (especially fringe parties) may develop flagship policies, or that policies change over time once elected – but a comprehensive understanding of what policies or pledges written in manifestos mean in a holistic manner is more useful to both journalist and voters than the pledges in manifestos alone. This is the purpose of the paper. Coalition governments are common in many countries, but not in the UK, which is the focus of this paper.*

Reviewer #5: This paper “creates a complex system model of the economic, societal and environmental landscape of the UK and assesses how it would be affected if political parties enacted their 2024 general election manifesto policies in full.”

This is an interesting and unique paper (I have not reviewed a paper like this before). This is largely a thought piece that explores important counterfactuals – what might happen if part X enacted its manifesto. Though it cannot ultimately test these counterfactuals with real world empirical evidence, I think this paper is worth publishing as long as there is an openness about the study’s limitations. And I think the authors are sufficiently open about its limitations, though I would recommend a little more of such openness towards the beginning of the paper.

This paper is proposing scenarios that are very unlikely (e.g., minor parties taking full control of the UK). Nonetheless, the paper is well-written and provides many useful insights. It uses innovative methods and encourages social scientists to think about the complex connections among concepts (especially second order effects).

So let me re-emphasize that the key is to be upfront about the paper's limitations, particularly at the beginning of the paper.

*We thank the reviewer for the comments. We agree, there are limits to the paper, especially, as included in response to reviewer 3, changes do occur when in government, compared to what is written in the manifesto. As indicated above, some of the paper focusses on what manifesto pledges mean* if *enacted, and show how some pledges which may appeal to voters (e.g. reduction of income tax), may in fact result in less overall income, especially into poorer households. We have made changes to the introduction to address the reviewer’s concerns (lines 97-101).*

Reviewer #6: As I am reviewing a revised version of the manuscript along with the earlier reviews, I see that authors put effort in making substantial changes in the manuscript which seems to lead an improved version. These changes also address some concerns I might have had. However, the lack of code and the opportunity to check it for reproducibility purposes makes it hard to arrive a conclusion even though majority of the work done with a huge undertaking.

*We thank the reviewer for the positive comments on the manuscript. The work should have been reproducible using the data provided, but to further improve reproducibility, we have added the R script and all necessary files to reproduce the sensitivity analysis and the model outcomes. It should be noted that as aspects of the model are based on random selection of values, sensitivity analysis may vary from that shown in the paper. The overall model results should be identical.*

Reviewer #7: Review of Manuscript: Evaluating Post-2024 Election Scenarios for the UK Based on Political Party Manifestos
The manuscript explores the application of a complex systems model to assess the holistic impact of UK political party manifestos for the 2024 general election. The main claim is that this modeling approach reveals both direct and indirect consequences of manifesto policies that are often obscured or misrepresented in political rhetoric. Notably, it finds that parties advocating strong tax cuts may inadvertently contribute to lower average incomes and higher inequality — a significant and counterintuitive insight.

The study is conceptually relevant and timely, particularly in the context of increasing demand for more transparent, evidence-based policy analysis. It offers a novel approach with potential utility for voters, policymakers, and political analysts.

The rationale for the study is well-articulated, and the abstract clearly identifies the gap the authors aim to address — namely, the disconnect between stated manifesto goals and actual systemic impacts. While I am not an expert in this specific modeling approach, the framing of manifestos as complex, interdependent political texts is sound and reflects current thinking in political communication and electoral studies.

*We thank the reviewer for these positive comments.*

That said, it would be useful for the authors to clarify how their model builds upon or diverges from previous approaches that attempt to quantify or simulate manifesto promises (e.g., spatial models, sentiment analysis, or policy simulation tools).

*The approach taken by our model is quite different from spatial positioning and sentiment analysis, but similar in many respects to some policy simulation tools. In the modified introduction, we mention policy simulation tools and previous studies – largely these tend to focus on one outcome area (lines 87-90). As the approach is quite different, we do not mention the other techniques until the discussion. Spatial positioning, and how our model builds on and differs from this is addressed in section 4.3. We have added a brief section on sentiment analysis (lines 514-518), which we think may prove useful as part of a combined approach in future work.*

Spatial positioning – section 4.3

The manuscript is generally well-written, but I believe its readability for non-specialists should be improved. While the conclusions are somewhat lengthy, they are plausible and thought-provoking. However, the methodology section is difficult to follow in its current form. Specifically:

1) The steps taken to construct and run the model could be better explained, ideally with a synthetic visual representation (e.g., a flowchart or diagram summarizing key stages).

*This seems like a very sensible suggestion. We have added a flow diagram summarising the process as Figure 1. Within the diagram, it refers to the relevant sections in the methods which may provide more detail.*

2) The data sources beyond the manifestos themselves are unclear. A summary table listing all data types, sources, and uses would enhance transparency and help readers better assess the robustness of the analysis.

*We hope the figure above helps clarify this. The manifestos provide the key data in terms of policy levers and outcomes. Other than this, little data (at least in the traditional sense) are needed. The model is constructed and initially parameterised through an understanding of the* direct *interactions between the model nodes. Much of this was the subject of discussion between the authors, but as the model considers only direct links between nodes, most of these will be relatively intuitive. The weighting of the interactions or ‘edges’ is also kept simple (ranging from 1-4, or weak to very strong). It is true that subjectivity can occur in the links and weightings, but the use of the large discussion group helps prevent this. Finally, the most important edge strengths are identified through sensitivity analysis, and checked, where possible, against relevant literature. While obtaining a final score from literature can be subjective, guidelines are provided in the model methods paper (Dominguez Almela et al. 2024). The literature used is provided in the table in supplementary material 1.*

*As such, we do understand why data sources are not obvious in the paper. However, it is a strength of the technique that we can model such complex systems with minimal data requirements.*

3)The selection of manifesto topics included in the model seems potentially arbitrary, notwithstanding the authors’ efforts to justify it. Why didn’t they consider topic modelling, at least for validation purposes?

*We were not aware of Topic Modelling when conducting the study, and we agree it could prove useful in the future, and indeed, potentially make studies such as these simpler to complete. We thank the reviewer for this suggestion. The topics selected in this study are not really a subset of topics from the manifestos, but a comprehensive list of the topics covered in the manifestos, along with intermediate nodes/topics which are necessary to construct the cause-and-effect relationships between these nodes. We appreciate this results in some strange outcomes, for example National Service was only included in the Conservative manifesto, hence that manifesto clearly had the best outcome for this node. However, national service can interact with other nodes, such as education and employment, and removing this topic would result in an incomplete holistic picture of the effect of this manifesto. If topics do not look complete, this may be due to ‘lumping’ some aspects together. For example, National Insurance was largely considered under income tax, as the effect throughout the model would be identical (this is detailed in Table 1). This ‘lumping’ of topics was necessary to reduce the complexity of the model, but has limited effects on the model outcome.*

Addressing these points would go a long way toward reinforcing the credibility of the conclusions.

I believe the manuscript has potential and is generally suitable for publication in PLOS ONE, pending minor-to-moderate revisions aimed at improving clarity and transparency. As I am reviewing this in the second round and was not involved in earlier stages, I cannot speak to prior revisions, but the current version would certainly benefit from clearer presentation of methods and data.
